# Narrative review of the safety and efficacy of marijuana for the treatment of commonly state-approved medical and psychiatric disorders

**DOI:** 10.1186/s13722-015-0032-7

**Published:** 2015-04-21

**Authors:** Katherine A Belendiuk, Lisa L Baldini, Marcel O Bonn-Miller

**Affiliations:** Institute of Human Development, University of California, 1121 Tolman Hall #1690, Berkeley, CA 94720 USA; Palo Alto University, 1791 Arastradero Road, Palo Alto, CA 94304 USA; Center of Excellence in Substance Abuse Treatment and Education, Philadelphia VA Medical Center, 3900 Woodland Avenue, Philadelphia, PA 19104 USA; Center for Innovation to Implementation and National Center for PTSD, VA Palo Alto Health Care System, 795 Willow Road (152-MPD), Menlo Park, CA 94025 USA; Department of Psychiatry, University of Pennsylvania Perelman School of Medicine, 3440 Market Street, Philadelphia, PA 19104 USA

**Keywords:** Cannabis, Medical marijuana, Marijuana, Medicine, Treatment, Alzheimer’s disease, ALS, Cachexia, Cancer, Crohn’s disease, Epilepsy, Seizures, Glaucoma, Hepatitis C virus, HCV, HIV, AIDS, Multiple sclerosis, MS, Pain, Nausea, Vomiting, Post-traumatic stress disorder, PTSD

## Abstract

The present investigation aimed to provide an objective narrative review of the existing literature pertaining to the benefits and harms of marijuana use for the treatment of the most common medical and psychological conditions for which it has been allowed at the state level. Common medical conditions for which marijuana is allowed (i.e., those conditions shared by at least 80 percent of medical marijuana states) were identified as: Alzheimer’s disease, amyotrophic lateral sclerosis, cachexia/wasting syndrome, cancer, Crohn’s disease, epilepsy and seizures, glaucoma, hepatitis C virus, human immunodeficiency virus/acquired immunodeficiency syndrome, multiple sclerosis and muscle spasticity, severe and chronic pain, and severe nausea. Post-traumatic stress disorder was also included in the review, as it is the sole psychological disorder for which medical marijuana has been allowed. Studies for this narrative review were included based on a literature search in PsycINFO, MEDLINE, and Google Scholar. Findings indicate that, for the majority of these conditions, there is insufficient evidence to support the recommendation of medical marijuana at this time. A significant amount of rigorous research is needed to definitively ascertain the potential implications of marijuana for these conditions. It is important for such work to not only examine the effects of smoked marijuana preparations, but also to compare its safety, tolerability, and efficacy in relation to existing pharmacological treatments.

## Introduction

National estimates suggest that 5.4 million people in the United States above the age of 12 have used marijuana daily or regularly within the past year [1]. This represents an increase of approximately 74.2 percent since 2006 [1]. Similar increases have also been noted among vulnerable populations in the U.S. (e.g., veterans and adolescents) [2,3].

Marijuana is currently illegal in every country in the world. In 2012, Uruguay voted to legalize state-controlled marijuana sales but implementation of the law has been postponed until 2015. The policy in the Netherlands is mixed, with permissible retail sale of marijuana at coffee shops, but restrictions on production and possession. Notably, as the concentration of THC in marijuana has increased, Dutch coffee shops have begun to close, as perception of marijuana as a “soft” drug transitions to perceptions of marijuana as a “hard” drug.

Like the Netherlands, the United States currently has a mixed drug policy; marijuana is an *illegal* Schedule I drug under U.S. Federal law. However, marijuana policies vary by state, with some states (e.g., Colorado and Washington) *legalizing* the use of recreational marijuana (i.e., allowing the legal possession and use of marijuana under state law), and other states *decriminalizing* marijuana (i.e., reducing the penalties for possession and/or use of small amounts of marijuana to fines or civil penalties). Furthermore, as of this review, 23 states and the District of Columbia have passed legislation allowing medical marijuana (i.e., individuals can defend themselves against criminal charges related to marijuana possession if a medical need is documented) for the treatment of a variety of medical and psychological conditions. Though the list of conditions for which medical marijuana has been allowed varies at the state level, the majority of states agree on its use for Alzheimer’s disease (AD), amyotrophic lateral sclerosis (ALS), cachexia/wasting syndrome, cancer, Crohn’s disease (CD), epilepsy and seizures, glaucoma, hepatitis C virus (HCV), human immunodeficiency virus (HIV)/acquired immunodeficiency syndrome (AIDS), multiple sclerosis (MS) and muscle spasticity, severe and chronic pain, severe nausea, and post-traumatic stress disorder (PTSD).

The aim of the present review is to provide a summary of the existing empirical literature regarding the effects of marijuana/cannabinoids on each of the above-noted conditions. Though some recent work has reviewed the adverse effects of marijuana [4] or the efficacy of marijuana for certain conditions (e.g., neurologic) [5], there has yet to be a comprehensive review of the effects of marijuana for each of the medical and psychiatric conditions for which it is currently used.

## Methods

The list of all conditions for which medical marijuana is allowed, according to the legislation of each U.S. state for which medical marijuana has been approved, was obtained and examined [6]. From this list, common conditions for which medical marijuana is allowed (i.e., those conditions shared by at least 80 percent of medical marijuana states) were identified as: AD, ALS, cachexia/wasting syndrome, cancer, CD, epilepsy and seizures, glaucoma, HCV, HIV/AIDS, MS and muscle spasticity, severe and chronic pain, and severe nausea. Though not presently a qualifying condition in at least 80 percent of states with medical marijuana laws, PTSD was also included in the review, as it is rapidly gaining attention and recognition as the sole psychological disorder for which medical marijuana is allowed.

Studies for this narrative review were included based on a literature search in the following databases: PsycINFO, MEDLINE, and Google Scholar. Within each database, each combination of the following key marijuana terms and the above-listed conditions were used to conduct a search: cannabis, marijuana, marihuana, cannabinoid, delta-9-tetrahydrocannabinol, THC, cannabidiol, CBD, cannabinol, cannabigerol, Marinol, dronabinol, Sativex, Nabilone, and Nabiximols. References within each obtained article were also examined to assure that no studies were overlooked. Only published, English-language studies were included in this review.

Though the primary focus of this review is on studies of marijuana plant effects, as these are most relevant to recent medical marijuana legislation, synthetic or plant-derived cannabinoids (e.g., dronabinol, Nabilone) were also included due to the general dearth of marijuana plant studies for a number of conditions. Indeed, for purposes of the review, references to oral administrations of marijuana constitute a pharmaceutical grade extraction administered in tablet or liquid form (e.g., dronabinol, Nabilone, Nabiximols), while references to smoked administration of marijuana constitute the inhalation of smoke from burned marijuana leaves and flowers. Finally, the present review is organized alphabetically by condition for which marijuana is allowed, rather than in order of disorder for which it is most to least commonly recommended, or strength of the evidence. We chose this approach as there is currently only state-level data [7-9], rather than national, representative data on the primary conditions for which medical marijuana is used or recommended, and the existing literature and state of the evidence for many conditions remains relatively poor.

## Results

### Alzheimer’s disease

AD, the leading form of dementia in the elderly, is a progressive, age-related disorder characterized by cognitive and memory deterioration [10]. AD has several neuropathological markers, including neuritic plaques and neurofibrillary tangles [11]. Although several researchers have suggested dronabinol and Nabilone may act on these mechanisms to confer therapeutic effects for patients with AD [12,13], a recent Cochrane systematic review found no evidence that dronabinol was effective in reducing symptoms of dementia [14]. The authors of a placebo-controlled crossover study of 15 patients with AD who were refusing to eat suggest that dronabinol increases weight gain and decreases disturbed behavior [15], but there is insufficient quantitative data to support this conclusion [14], and one study participant had a grand mal seizure following dronabinol administration [15]. Another pilot study of two patients with dementia found that dronabinol reduced nocturnal motor activity [16]. No studies have examined the effects of smoked marijuana in patients with AD. In sum, there is insufficient evidence to recommend marijuana for the treatment of AD. Future directions should include conducting randomized controlled trials (RCTs) comparing both smoked and oral marijuana to placebo and existing treatments, with sample sizes large enough to detect treatment effects and the safety and tolerability of marijuana.

### Amyotrophic lateral sclerosis

ALS is a fatal neurological disease with symptoms that include weakness, spasticity, and respiratory difficulties. Cannabinoids are hypothesized to act in the regions of established pathophysiology for ALS [17] and could be used for symptom management (e.g., pain, spasticity, wasting, respiratory failure, dysphagia, negative mood, and dysautonomia) [18]. Although there is limited evidence from a survey of patients with ALS that marijuana consumed in a variety of forms (i.e., oral, smoked, vaporized, and eaten) improves speech and swallowing [19], the anti-salivatory components of marijuana may reduce the risk of aspiration pneumonia, while also increasing patient comfort [18,19]. These survey findings indicate that up to 10 percent of patients use marijuana for symptom management, and these self-reports suggest efficacy in increasing appetite and mood and decreasing pain, spasticity, and drooling. However, as is consistent with the half-life of smoked marijuana, the beneficial effects of marijuana on symptoms of ALS were fewer than 3 hours in duration [19]. The only randomized, double-blind, placebo-controlled crossover trial of marijuana in patients with ALS has a small sample size (N = 27) and indicates that while 5 mg of dronabinol is well-tolerated, there was no effect on number or intensity of cramps, quality of life, appetite, sleep, or mood [20]. There is currently insufficient clinical evidence in humans with ALS to recommend cannabinoids as primary or adjunctive therapy.

### Cachexia/wasting syndrome

Cachexia is the general wasting and malnutrition that occurs in the context of chronic diseases such as HIV/AIDS and cancer. In patients with HIV or cancer, smoked marijuana and dronabinol have been shown to increase weight gain [21,22] and food intake [22,23] compared to placebo. In a within-subject, double-blind, staggered, double-dummy study of nine individuals with muscle mass loss, dronabinol resulted in significantly greater calorie consumption than smoked marijuana [24]. A within-subject, double-blind, placebo-controlled trial with seven HIV-positive marijuana smokers taking antiretroviral medications found that compared to placebo, dronabinol increased caloric intake [25]. Additional studies indicate that dronabinol administration increases appetite, decreases nausea, and protects against weight loss [26], with effects on appetite and weight stability enduring in long-term follow-up [27].

Both dronabinol and smoked marijuana increase the number of eating occasions [22,25], and smoked marijuana may also affect weight gain and calorie intake by modulating appetite hormones [28]. Importantly, weight gain in one study was greater than would have been expected based on increased calorie consumption alone [23], which may be particularly relevant for those who have impaired food intake and/or nausea. These studies demonstrate that marijuana has positive effects on cachexia resulting from a medical condition, but are largely limited by small sample sizes. Additionally, studies comparing THC to FDA-approved medication (i.e., megestrol) indicate that THC is less effective in promoting appetite and weight gain [29]. In sum, there is moderate support for the use of cannabinoids for cachexia/wasting, and dronabinol has been FDA-approved for anorexia associated with weight loss in individuals with AIDS. Additional studies with larger sample sizes that examine the efficacy of marijuana compared to nutritional support/calorie augmentation in the treatment of cachexia are indicated.

### Cancer

Cancer is a qualifying medical condition in every state that has approved marijuana for medical use [30]. The majority of clinical research examining the relation between THC and cancer has evaluated the effect of smoked THC on the risk for cancer, or the palliative effects of THC on chemotherapy-related nausea and emesis, chronic pain, and wasting (reviewed in respective sections); few studies have studied the effect of marijuana in any form on the treatment of primary cancer pathology. *In vitro* and *in vivo* research suggests that cannabinoids inhibit tumor growth [30] via several proposed mechanisms (e.g., suppression of cell proliferation, reduced cell migration, increased apoptosis) [31]; however, *in vitro* and *in vivo* studies also have shown that THC increases tumor growth due to reduced immune response to cancer [32]. The only clinical trial of THC on cancer examined intracranial administration of THC to nine patients with recurrent glioblastoma multiforme who had failed surgical- and radiotherapy, and results indicated that THC decreased tumor growth, while being well-tolerated with few psychotropic effects [33]. This study is limited by lack of generalizability, and clinical trials with larger representative samples that examine oral or smoked administration of THC are essential to elucidate the effects on cancer pathology. There is currently insufficient evidence to recommend marijuana for the treatment of cancer, but there may be secondary treatment effects on appetite and pain.

### Crohn’s disease

CD is an inflammatory bowel disease (IBD) that has no cure; treatment targets include reducing inflammation and secondary symptoms. Between 16 percent and 50 percent of patients use marijuana to relieve symptoms of IBD [34-36], and patients using marijuana for 6 months or longer are five times more likely to have had surgery for their IBD [34]; whether marijuana exacerbates disease progression or more severe disease results in self-medication is unclear. Only one placebo-controlled study of the effects of marijuana in patients with CD has been conducted [37]. This study found that there was no difference between placebo and smoked marijuana on CD remission (defined as a CD Activity Index (CDAI) of less than 100), and that marijuana was superior to placebo in promoting clinical response (a decrease in CDAI score greater than 100), reducing steroid use, and improving sleep and appetite [37]. Importantly, this study did not include objective measurement of inflammatory activity, and there was no significant difference in placebo and treatment groups 2 weeks after treatment cessation [37]. Until clinical trials with objective measurement of treatment effects over an extended period of time are conducted to examine the safety and efficacy of marijuana for the treatment of IBD, there is insufficient evidence for the use of marijuana for the treatment of IBD.

### Epilepsy and seizures

The known effects of cannabinoids on epilepsy and seizures are largely from animal studies, surveys, and case studies. Several animal studies indicate that marijuana and its constituents exhibit anticonvulsant effects [38-41] and reduce seizure-related mortality [39], but there is also evidence that cannabinoids can lower the threshold for seizures [42], and THC withdrawal increases susceptibility for convulsions [42]. Cross-sectional surveys indicate that 16–21 percent of patients with epilepsy smoke marijuana [43,44], with some reporting positive effects (e.g., spasm reduction) and a belief that marijuana is an effective therapy [44], and others reporting increased seizure frequency and intensity [43]. Based on a Cochrane review, the few RCTs that have been conducted in humans include a total of 48 participants [45] and only examine treatment with cannabidiol. These trials exhibited heterogeneity of effects: some indicated a reduction in seizure frequency [46,47], while others demonstrated no effect compared to placebo [48]. In addition, none of the studies examined response at greater than 6-month follow-up [45]. Systematic reviews of the literature have concluded that there is insufficient clinical data to support or refute the use of cannabinoids for the treatment of epilepsy and seizures [5,45].

### Glaucoma

Glaucoma is a neurodegenerative eye disease that can cause blindness by damaging retinal ganglion cells and axons of the optic nerve. Intraocular pressure (IOP) can influence both onset and progression of glaucoma and is often a target for intervention. Small samples have demonstrated reduced IOP following smoked marijuana [49,50], but the effect is only present in 60–65 percent of individuals [51] and lasts for 3–4 hours, requiring repeated dosing throughout the day [52]. Furthermore, patients discontinue marijuana use due to side effects (e.g., dizziness, anxiety, dry mouth, sedation, depression, confusion, weight gain, and distortion of perception [53]), and this treatment discontinuity may exacerbate optic nerve damage and obviate the benefits of reduced IOP [54]. Limited research and documented toxicity have resulted in the American Glaucoma Society [54], Canadian Opthalmological Society [55], and the American Academy of Ophthalmology’s Complementary Therapies Task Force [52] determining that there is insufficient evidence to indicate that marijuana is safer or more effective than existing pharmacotherapy or surgery for the reduction of IOP. Development of eye drops for topical application of THC would minimize psychoactive and other side effects but is complicated by the high lipophilicity and low water solubility of cannabinoids [52,56]. Additionally, the distance from the application site to the retina may be too great to afford neuroprotective benefits [52], given that only 5 percent of an applied dose penetrates the cornea to the intraocular space [56].

### Hepatitis C virus

There have been no RCTs examining the use of cannabinoids on HCV infection. Of the studies that have been conducted, one longitudinal study demonstrates that smoked marijuana has no effect on HCV progression in individuals with HIV [57]. In contrast, individuals with HCV who smoke marijuana have a higher fibrosis progression rate [58] and more severe steatosis [59], with daily smokers having a more rapid rate of progression and greater severity [60] than occasional marijuana users [58,59]. Marijuana may have independent negative effects on steatosis [59], but because none of these findings were in the context of a clinical trial, these correlations are not causal and it is possible that individuals who use marijuana do so to manage greater symptom severity [60].

There may be secondary effects of cannabinoids on HCV treatment side effects: dronabinol and Nabilone stabilized treatment-induced weight-loss [61]; and dronabinol, Nabilone, and marijuana procured from a marijuana club (dose and method of administration unspecified) increased HCV treatment duration and reduced post-treatment virological relapse [61,62]. However, there is also a potential drug-drug interaction between ribavirin, a traditional HCV treatment, and marijuana due to shared cytochrome 450 metabolism [63]. Because 90 percent of HCV infections are the result of injection drug use [64], treatment of symptoms with marijuana may be contraindicated for this subpopulation, particularly because marijuana use in the context of other substance use (i.e., alcohol) has multiplicative effects on the odds of fibrosis severity [60]. Given that newer treatments for HCV (e.g., sofosbuvir) are replacing ribavirin, there will likely be less need for use of marijuana in management of treatment-related side effects. In sum, there is currently insufficient empirical support to recommend marijuana for the treatment of HCV.

### HIV/AIDS

Marijuana use in HIV-infected patients is typically for the management of side effects (e.g., nausea) of older antiretroviral treatments and AIDS-related symptoms, including weight-loss and HIV-associated neuropathy (covered in cachexia and pain sections, respectively). Survey studies indicate that 23 percent of patients with HIV/AIDS smoked marijuana in the past month and do so largely to improve mood and appetite and reduce pain [65]; these patients may exhibit tolerance and need higher doses of THC than are currently approved by the FDA for use in clinical trials [25] to experience treatment effects. The few RCTs that have been conducted in a small number of patients with HIV/AIDS largely examined the effects of marijuana (synthetic or natural marijuana that is smoked or ingested) on symptoms (e.g., nausea and appetite) over a short treatment window (21–84 days; see [66] for systematic review). Studies examining the effects of marijuana on the pharmacokinetics of antiretroviral medication demonstrated that neither smoked marijuana nor dronabinol affects short-term clinical outcomes (e.g., viral load, CD4 and CD8 counts [67]), influences the efficacy of antiretroviral medication [68], or indicates that dose adjustments for protease inhibitors are necessary [21]. However, individuals who are dependent on marijuana have demonstrated poorer medication adherence and greater HIV symptoms and side effects than nonusers and nondependent users [69]. Furthermore, while some studies have no participant withdrawal due to adverse events [21,70,71], others reported treatment-limiting adverse events [26,72,73]. Finally, because drug use is a risk factor for HIV infection [74], treatment of symptoms with marijuana may be contraindicated for this subpopulation. In sum, there is variability in short-term outcomes and insufficient long-term data addressing the safety and efficacy of marijuana when used to manage symptoms of HIV/AIDS and its role in those also using newer, better-tolerated antiretroviral agents.

### Multiple sclerosis and muscle spasticity

Muscle spasticity, a common feature of MS, is disordered sensorimotor control that leads to involuntary muscle activation [75] that results in pain, sleep disturbance, and increased morbidity [76]. The majority of studies examining spasticity have compared oral or sublingual forms of cannabinoids to placebo and found reduced spasm severity [77-84], with symptom improvement enduring at long-term follow-up [85-87], and also reduced spasm frequency and spasm-related pain and sleep disturbances [77,88,89]. With regard to smoked marijuana, one study found reductions in muscle spasticity [90]; however, another study showed that smoking marijuana impaired posture and balance in individuals with spasticity [91], so there is currently insufficient evidence to determine the efficacy of smoked marijuana on spasticity [5].

Surveys of patient populations show that between 14 and 16 percent of patients with MS report using marijuana for symptom management [92,93] and that compared to non-marijuana-using individuals with MS, marijuana-using individuals with MS have decreased cognitive functioning [90,94,95]. Because cognitive dysfunction is present in 40–60 percent of individuals with MS before marijuana administration [96], marijuana use may further compromise impaired cerebral functioning in a neurologically vulnerable population. Additionally, future studies should carefully consider outcome assessment. The primary methods of measuring spasticity, the Ashworth Scale and patient self-report, may not be appropriate measures because antispastic drugs do not decrease Ashworth ratings, and patient-reported spasticity severity may be poorly correlated with patient functioning (i.e., a patient whose spasticity compensated for motor weakness may be unable to ambulate with reduced spasticity) [97]. Importantly for both MS and other neurological disorders, the American Academy of Neurology does not advocate the use of marijuana for the treatment of neurological disorders, due to insufficient evidence regarding treatment efficacy [98].

### Post-traumatic stress disorder

There has been a recent emergence of empirical studies of the effects of marijuana on symptoms of PTSD, borne primarily out of the observation that individuals with PTSD report using marijuana to cope with PTSD symptoms; specifically, hyperarousal, negative affect, and sleep disturbances [99-101]. Empirical work has consistently demonstrated that the endocannabinoid system plays a significant role in the etiology of PTSD, with greater availability of cannabinoid type 1 receptors documented among those with PTSD than in trauma-exposed or healthy controls [102,103]. Though the use of marijuana and oral THC [104,105] have been implicated as a potential mechanism for the mitigation of many PTSD symptoms by way of their effects on the endocannabinoid system, some researchers caution that endocannabinoid activation with plant-based extracts over extended periods may lead to a number of deleterious consequences, including receptor downregulation and addiction [102].

There have been no RCTs of marijuana for the treatment of PTSD, though there has been one small RCT of Nabilone that showed promise for reducing nightmares associated with PTSD [106]. One unpublished pilot study of 29 Israeli combat veterans showed reductions in PTSD symptoms following the administration of smoked marijuana, with effects seen up to one year post-treatment [107]. Remaining studies have been primarily observational in nature, documenting that PTSD is associated with greater odds of a cannabis use disorder diagnosis [108] and greater marijuana craving and withdrawal immediately prior to a marijuana cessation attempt [109]. Indeed, sleep difficulties (a hallmark of PTSD) have been associated with poor marijuana cessation outcomes [110,111], while cannabis use disorders have been associated with poorer PTSD treatment outcomes [112]. Given the lack of RCTs studying marijuana as a treatment for PTSD, there is insufficient scientific evidence for its use at this time.

### Severe and chronic pain

Clinical trials have examined smoked and oral administration of cannabinoids on different types of pain (e.g., neuropathic, post-operative, experimentally induced) in multiple patient populations (e.g., HIV, cancer, and fibromyalgia). Two meta-analyses have been conducted examining the association between marijuana and pain. In the first, 18 RCTs demonstrated that any marijuana preparation containing THC, applied by any route of administration, significantly decreased pain scores from baseline compared to placebo [113]. The second examined 19 RCTs of smoked marijuana in individuals with HIV, which also indicated greater efficacy in reducing pain (i.e., sensory neuropathy) compared to placebo [114]. Importantly, the first meta-analysis showed that marijuana increased the odds of altered perception, motor function, and cognition by 4 to 5 times [113], and the second study did not recommend marijuana as routine therapy [114]. Dosage is an important factor to consider for administration of cannabinoids for pain management, as some studies have found that higher doses of smoked marijuana are associated with improved analgesia [115], whereas other studies show that higher doses of smoked marijuana increase pain response [116]. Because the analgesic effects of marijuana are comparable to those of traditional pain medications [117], future research should aim to identify which analgesics provide the lowest risk profile for the management of severe and chronic pain. Although there is preliminary support to suggest that marijuana may have analgesic effects, there is insufficient research on dosing and side effect profile, which precludes recommending marijuana for the management of severe and chronic pain.

### Severe nausea

The majority of research related to the effects of marijuana on severe nausea has involved oral administration of marijuana to individuals with chemotherapy-induced nausea and vomiting (CINV). Oral marijuana (i.e., THC suspension in sesame oil and gelatin) has been shown to be more effective in reducing CINV than placebo [118], including the number and volume of vomiting episodes, and the severity and duration of nausea [119]. When compared to traditional anti-emetics, some meta-analytic reviews indicate that oral THC is more effective in reducing CINV [120-123], others find no significant difference [122,124-126], and another suggests that combining both is the most effective at reducing the duration and severity of CINV than either alone [127]. Recent advances in both anti-emetic agents and the mechanisms of cannabinoid administration (i.e., sublingual application) warrant future research.

Importantly, patients receiving cannabinoids for severe nausea reported toxicities, including paranoid delusions (5%), hallucinations (6%), and dysphoria (13%) [122]. Additionally, cannabinoid hyperemesis syndrome has been documented, in which persistent and regular marijuana use (i.e., daily or weekly use for more than 1 year) is associated with cyclic vomiting (i.e., episodic nausea and vomiting) [128] and nonresponse to treatment for cyclic vomiting [129]. Dronabinol has been FDA-approved for CINV in individuals who have not shown a treatment response to traditional anti-emetics, but in line with recommendations from the American Society of Clinical Oncology [130] and the European Society for Medical Oncology [131], cannabinoids should not be utilized as a first-line treatment for nausea and vomiting.

## Conclusions

The reviewed literature highlights the dearth of rigorous research on the effects of marijuana for the most common conditions for which it is currently recommended. It is paramount that well-designed RCTs with larger sample sizes be conducted to determine the actual medical benefits and adverse effects of marijuana for each of the above conditions. Indeed, recent reviews [4,132] comprehensively discuss adverse events associated with marijuana use, and while it is beyond the scope of the current paper to review these effects in-depth, they are important to consider when evaluating whether or not to recommend marijuana for a medical or psychiatric disorder in place of other existing treatment options.

Given the extensive literature speaking to the harms associated with marijuana use, research on the comparative safety, tolerability, efficacy, and risk of marijuana compared to existing pharmacological agents is needed. The present literature also illuminates the need for research into the effects of isolated cannabinoids (e.g., THC, CBD) as well as species of smoked marijuana (e.g., indica and sativa), as the majority of medical marijuana users ingest marijuana by smoking the marijuana plant [133,134], which contains a wide variety of phytocannabinoids at varying potencies [135,136]. Furthermore, improved and objective measurement of clinical outcomes should be implemented in clinical trials to determine treatment efficacy. Finally, little research has considered the issues of dose, duration, and potency. If research identifies a therapeutic effect of marijuana for medical or psychiatric conditions, there will need to be revisions in marijuana policy to increase quality control so that dose and potency are valid and reliable. Additionally, risk of abuse and diversion can be decreased by developing prescribing practices with continued supervision of a medical professional, creating prescription monitoring programs to reduce the risk of “doctor shopping”, and identifying provisions for the safe disposal of unused cannabinoids. In sum, the current literature does not adequately support the widespread adoption and use of marijuana for medical and psychiatric conditions at this time.

## Abbreviations

THC: Δ^9^-tetrahydrocannabinol
HIV: Human immunodeficiency virus
AIDS: Acquired immunodeficiency syndrome
RCTs: Randomized controlled trials
IOP: Intraocular pressure
MS: Multiple sclerosis
CINV: Chemotherapy-induced nausea and vomiting
HCV: Hepatitis C virus
ALS: Amyotrophic lateral sclerosis
CD: Crohn’s disease
IBD: Inflammatory bowel disease
AD: Alzheimer’s disease
PTSD: Post-traumatic stress disorder
CB1: Cannabinoid type 1
CBD: Cannabidiol

## References

[1] Substance Abuse and Mental Health Services Administration (2013). Results from the 2012 National Survey on Drug Use and Health: Summary of National Findings.

[2] Bonn-Miller MO, Harris AH, Trafton JA (2012). Prevalence of cannabis use disorder diagnoses among veterans in 2002, 2008, and 2009. Psycholog Serv.

[3] Johnston LD, O'Malley PM, Miech RA, Bachman JG, Schulenberg JE (2014). Monitoring the Future national results on drug use: 1975–2013: Overview, Key Findings on Adolescent Drug Use.

[4] Volkow ND, Baler RD, Compton WM, Weiss SR (2014). Adverse health effects of marijuana use. N Engl J Med.

[5] Koppel BS, Brust JC, Fife T, Bronstein J, Youssof S, Gronseth G (2014). Systematic review: efficacy and safety of medical marijuana in selected neurologic disorders: report of the guideline development subcommittee of the American academy of neurology. Neurology.

[6] Procon.org. 23 legal medical marijuana states and DC: Laws, fees, and possession limits. Retrieved April 9, 2015, from [http://medicalmarijuana.procon.org/view.resource.php?resourceID=000881]

[7] Bonn-Miller MO, Boden MT, Bucossi MM, Babson KA (2014). Self-reported cannabis use characteristics, patterns and helpfulness among medical cannabis users. Am J Drug Alcohol Abuse.

[8] Ilgen MA, Bohnert K, Kleinberg F, Jannausch M, Bohnert ASB, Walton MA (2013). Characteristics of adults seeking medical marijuana certification. Drug Alcohol Depend.

[9] Nunberg H, Kilmer B, Pacula RL, Burgdorf J (2011). An analysis of applicants presenting to a medical marijuana specialty practice in California. J Drug Policy Anal..

[10] Minati L, Edginton T, Bruzzone MG, Giaccone G (2009). Reviews: current concepts in Alzheimer's disease: a multidisciplinary review. Am J Alzheimers Dis Other Demen.

[11] Fagan SG, Campbell VA (2014). The influence of cannabinoids on generic traits of neurodegeneration. Br J Pharmacol.

[12] Gowran A, Noonan J, Campbell VA (2011). The multiplicity of action of cannabinoids: implications for treating neurodegeneration. CNS Neurosci Ther.

[13] Aso E, Ferrer I (2014). Cannabinoids for treatment of Alzheimer’s disease: moving toward the clinic. Front Pharmacol.

[14] Krishnan S, Cairns R, Howard R (2009). Cannabinoids for the treatment of dementia. Cochrane Database Syst Rev.

[15] Volicer L, Stelly M, Morris J, McLaughlin J, Volicer BJ (1997). Effects of dronabinol on anorexia and disturbed behavior in patients with Alzheimer's disease. Int J Geriatr Psychiatry.

[16] Walther S, Schüpbach B, Seifritz E, Homan P, Strik W (2011). Randomized, controlled crossover trial of dronabinol, 2.5 mg, for agitation in 2 patients with dementia. J Clin Psychopharmacol.

[17] Carter GT, Abood ME, Aggarwal SK, Weiss MD (2010). Cannabis and amyotrophic lateral sclerosis: hypothetical and practical applications, and a call for clinical trials. Am J Hosp Palliat Care.

[18] Carter GT, Rosen BS (2001). Marijuana in the management of amyotrophic lateral sclerosis. Am J Hosp Palliat Care.

[19] Amtmann D, Weydt P, Johnson KL, Jensen MP, Carter GT (2004). Survey of cannabis use in patients with amyotrophic lateral sclerosis. Am J Hosp Palliat Care.

[20] Weber M, Goldman B, Truniger S (2010). Tetrahydrocannabinol (THC) for cramps in amyotrophic lateral sclerosis: a randomised, double-blind crossover trial. J Neurol Neurosurg Psychiatry.

[21] Abrams DI, Hilton JF, Leiser RJ, Shade SB, Elbeik TA, Aweeka FT (2003). Short-term effects of cannabinoids in patients with HIV-1 infection: a randomized, placebo-controlled clinical trial. Ann Intern Med.

[22] Haney M, Gunderson EW, Rabkin J, Hart CL, Vosburg SK, Comer SD (2007). Dronabinol and marijuana in HIV-positive marijuana smokers: caloric intake, mood, and sleep. J Acquir Immune Defic Syndr.

[23] Foltin RW, Fischman MW, Byrne MF (1988). Effects of smoked marijuana on food intake and body weight of humans living in a residential laboratory. Appetite.

[24] Haney M (2002). Effects of smoked marijuana in healthy and HIV+ marijuana smokers. J Clin Pharmacol.

[25] Bedi G, Foltin RW, Gunderson EW, Rabkin J, Hart CL, Comer SD (2010). Efficacy and tolerability of high-dose dronabinol maintenance in HIV-positive marijuana smokers: a controlled laboratory study. Psychopharmacol (Berl).

[26] Beal JE, Olson R, Laubenstein L, Morales JO, Bellman P, Yangco B (1995). Dronabinol as a treatment for anorexia associated with weight loss in patients with AIDS. J Pain Symptom Manage.

[27] Beal JE, Olson R, Lefkowitz L, Laubenstein L, Bellman P, Yangco B (1997). Long-term efficacy and safety of dronabinol for acquired immunodeficiency syndrome-associated anorexia. J Pain Symptom Manage.

[28] Riggs PK, Vaida F, Rossi SS, Sorkin LS, Gouaux B, Grant I (2012). A pilot study of the effects of cannabis on appetite hormones in HIV-infected adult men. Brain Res.

[29] Jatoi A, Windschitl HE, Loprinzi CL, Sloan JA, Dakhil SR, Mailliard JA (2002). Dronabinol versus megestrol acetate versus combination therapy for cancer-associated anorexia: a north central cancer treatment group study. J Clin Oncol.

[30] Bowles DW, O’Bryant CL, Camidge DR, Jimeno A (2012). The intersection between cannabis and cancer in the United States. Crit Rev Oncol Hematol.

[31] Pisanti S, Malfitano AM, Grimaldi C, Santoro A, Gazzerro P, Laezza C (2009). Use of cannabinoid receptor agonists in cancer therapy as palliative and curative agents. Best Prac Res Clin Endocrinol Metab.

[32] McKallip RJ, Nagarkatti M, Nagarkatti PS (2005). Δ-9-tetrahydrocannabinol enhances breast cancer growth and metastasis by suppression of the antitumor immune response. J Immunol.

[33] Guzman M, Duarte MJ, Blazquez C, Ravina J, Rosa MC, Galve-Roperh I (2006). A pilot clinical study of Δ9-tetrahydrocannabinol in patients with recurrent glioblastoma multiforme. Br J Cancer.

[34] Storr M, Devlin S, Kaplan GG, Panaccione R, Andrews CN (2014). Cannabis use provides symptom relief in patients with inflammatory bowel disease but is associated with worse disease prognosis in patients with Crohn's disease. Inflamm Bowel Dis.

[35] Lal S, Prasad N, Ryan M, Tangri S, Silverberg MS, Gordon A (2011). Cannabis use amongst patients with inflammatory bowel disease. Eur J Gastroenterol Hepatol.

[36] Allegretti JR, Courtwright A, Lucci M, Korzenik JR, Levine J (2013). Marijuana use patterns among patients with inflammatory bowel disease. Inflamm Bowel Dis.

[37] Naftali T, Bar-Lev Schleider L, Dotan I, Lansky EP, Sklerovsky Benjaminov F, Konikoff FM (2013). Cannabis induces a clinical response in patients with Crohn's disease: a prospective placebo-controlled study. Clin Gastroenterol Hepatol.

[38] Hill TD, Cascio MG, Romano B, Duncan M, Pertwee RG, Williams CM (2013). Cannabidivarin-rich cannabis extracts are anticonvulsant in mouse and rat via a CB1 receptor-independent mechanism. Br J Pharmacol.

[39] Jones NA, Glyn SE, Akiyama S, Hill TD, Hill AJ, Weston SE (2012). Cannabidiol exerts anti-convulsant effects in animal models of temporal lobe and partial seizures. Seizure.

[40] Wallace MJ, Blair RE, Falenski KW, Martin BR, DeLorenzo RJ (2003). The endogenous cannabinoid system regulates seizure frequency and duration in a model of temporal lobe epilepsy. J Pharmacol Exp Ther.

[41] Jones NA, Hill AJ, Smith I, Bevan SA, Williams CM, Whalley BJ (2010). Cannabidiol displays antiepileptiform and antiseizure properties in vitro and in vivo. J Pharmacol Exp Ther.

[42] Gordon E, Devinsky O (2001). Alcohol and marijuana: effects on epilepsy and use by patients with epilepsy. Epilepsia.

[43] Hamerle M, Ghaeni L, Kowski A, Weissinger F, Holtkamp M (2014). Cannabis and other illicit drug use in epilepsy patients. Eur J Neurol.

[44] Gross DW, Hamm J, Ashworth NL, Quigley D (2004). Marijuana use and epilepsy: prevalence in patients of a tertiary care epilepsy center. Neurology.

[45] Gloss D, Vickrey B (2014). Cannabinoids for epilepsy. Cochrane Database Syst Rev.

[46] Mechoulam R, Carlini EA (1978). Toward drugs derived from cannabis. Naturwissenschaften.

[47] Cunha JM, Carlini EA, Pereira AE, Ramos OL, Pimentel C, Gagliardi R (1980). Chronic administration of cannabidiol to healthy volunteers and epileptic patients. Pharmacology.

[48] Ames FR, Cridland S (1986). Anticonvulsant effect of cannabidiol. S Afr Med J.

[49] Merritt JC, Crawford WJ, Alexander PC, Anduze AL, Gelbart SS (1980). Effect of marihuana on intraocular and blood pressure in glaucoma. Ophthalmology.

[50] Hepler RS, Frank IR (1971). Marihuana smoking and intraocular pressure. JAMA.

[51] Green K (1998). Marijuana smoking vs cannabinoids for glaucoma therapy. Arch Ophthalmol.

[52] Nucci C, Bari M, Spanò A, Corasaniti M, Bagetta G, Maccarrone M (2008). Potential roles of (endo) cannabinoids in the treatment of glaucoma: from intraocular pressure control to neuroprotection. Prog Brain Res.

[53] Flach AJ (2002). Delta-9-tetrahydrocannabinol (THC) in the treatment of end-stage open-angle glaucoma. Trans Am Ophthalmol Soc.

[54] Jampel H (2010). American glaucoma society position statement: marijuana and the treatment of glaucoma. J Glaucoma.

[55] Buys YM, Rafuse PE (2010). Canadian ophthalmological society policy statement on the medical use of marijuana for glaucoma. Can J Ophthalmol.

[56] Tomida I, Pertwee RG, Azuara-Blanco A (2004). Cannabinoids and glaucoma. Br J Ophthalmol.

[57] Brunet L, Moodie EE, Rollet K, Cooper C, Walmsley S, Potter M (2013). Marijuana smoking does not accelerate progression of liver disease in HIV-hepatitis C coinfection: a longitudinal cohort analysis. Clin Infect Dis.

[58] Hézode C, Roudot-Thoraval F, Nguyen S, Grenard P, Julien B, Zafrani E-S (2005). Daily cannabis smoking as a risk factor for progression of fibrosis in chronic hepatitis C. Hepatology.

[59] Hézode C, Zafrani ES, Roudot-Thoraval F, Costentin C, Hessami A, Bouvier–Alias M (2008). Daily cannabis use: a novel risk factor of steatosis severity in patients with chronic hepatitis C. Gastroenterology.

[60] Ishida JH, Peters MG, Jin C, Louie K, Tan V, Bacchetti P (2008). Influence of cannabis use on severity of hepatitis C disease. Clin Gastroenterol Hepatol.

[61] Costiniuk CT, Mills E, Cooper CL (2008). Evaluation of oral cannabinoid-containing medications for the management of interferon and ribavirin-induced anorexia, nausea and weight loss in patients treated for chronic hepatitis C virus. Can J Gastroenterol.

[62] Sylvestre DL, Clements BJ, Malibu Y (2006). Cannabis use improves retention and virological outcomes in patients treated for hepatitis C. Eur J Gastroenterol Hepatol.

[63] Glue P (1998). The clinical pharmacology of ribavirin. Semin Liver Dis.

[64] Wasley A, Grytdal S, Gallagher K, Centers for Disease Control and Prevention (CDC) (2008). Surveillance for acute viral hepatitis -- United States, 2006. MMWR Surveill Summ.

[65] Prentiss D, Power R, Balmas G, Tzuang G, Israelski DM (2004). Patterns of marijuana use among patients with HIV/AIDS followed in a public health care setting. J Acquir Immune Defic Syndr.

[66] Lutge EE, Gray A, Siegfried N (2013). The medical use of cannabis for reducing morbidity and mortality in patients with HIV/AIDS. Cochrane Database Syst Rev.

[67] Bredt BM, Higuera-Alhino D, Shade SB, Hebert SJ, McCune JM, Abrams DI (2002). Short-term effects of cannabinoids on immune phenotype and function in HIV-1-infected patients. J Clin Pharmacol.

[68] Kosel BW, Aweeka FT, Benowitz NL, Shade SB, Hilton JF, Lizak PS (2002). The effects of cannabinoids on the pharmacokinetics of indinavir and nelfinavir. AIDS.

[69] Bonn-Miller MO, Oser ML, Bucossi MM, Trafton JA (2014). Cannabis use and HIV antiretroviral therapy adherence and HIV-related symptoms. J Behav Med.

[70] Haney M, Rabkin J, Gunderson E, Foltin RW (2005). Dronabinol and marijuana in HIV(+) marijuana smokers: acute effects on caloric intake and mood. Psychopharmacology (Berl).

[71] Abrams DI, Jay CA, Shade SB, Vizoso H, Reda H, Press S (2007). Cannabis in painful HIV-associated sensory neuropathy: a randomized placebo-controlled trial. Neurology.

[72] Ellis RJ, Toperoff W, Vaida F, van den Brande G, Gonzales J, Gouaux B (2009). Smoked medicinal cannabis for neuropathic pain in HIV: a randomized, crossover clinical trial. Neuropsychopharmacology.

[73] Struwe M, Kaempfer SH, Geiger CJ, Pavia AT, Plasse TF, Shepard KV (1993). Effect of dronabinol on nutritional status in HIV infection. Ann Pharmacother.

[74] Centers for Disease Control and Prevention (CDC). HIV and substance use in the United States*.* Retrieved April 9, 2015, from [http://www.cdc.gov/hiv/risk/behavior/substanceuse.html]

[75] Pandyan AD, Gregoric M, Barnes MP, Wood D, van Wijck F, Burridge J (2005). Spasticity: clinical perceptions, neurological realities and meaningful measurement. Disabil Rehabil.

[76] Beard S, Hunn A, Wight J (2003). Treatments for spasticity and pain in multiple sclerosis: a systematic review. Health Technol Assess.

[77] Novotna A, Mares J, Ratcliffe S, Novakova I, Vachova M, Zapletalova O (2011). A randomized, double-blind, placebo-controlled, parallel-group, enriched-design study of nabiximols*(Sativex®), as add-on therapy, in subjects with refractory spasticity caused by multiple sclerosis. Eur J Neurol.

[78] Wade DT, Makela P, Robson P, House H, Bateman C (2004). Do cannabis-based medicinal extracts have general or specific effects on symptoms in multiple sclerosis? A double-blind, randomized, placebo-controlled study on 160 patients. Mult Scler.

[79] Collin C, Davies P, Mutiboko IK, Ratcliffe S (2007). Randomized controlled trial of cannabis-based medicine in spasticity caused by multiple sclerosis. Eur J Neurol.

[80] Wade DT, Collin C, Stott C, Duncombe P (2010). Meta-analysis of the efficacy and safety of Sativex (nabiximols), on spasticity in people with multiple sclerosis. Mult Scler.

[81] Wade DT, Robson P, House H, Makela P, Aram J (2003). A preliminary controlled study to determine whether whole-plant cannabis extracts can improve intractable neurogenic symptoms. Clin Rehabil.

[82] Zajicek JP, Sanders HP, Wright DE, Vickery PJ, Ingram WM, Reilly SM (2005). Cannabinoids in multiple sclerosis (CAMS) study: safety and efficacy data for 12 months follow up. J Neurol Neurosurg Psychiatry.

[83] Zajicek J, Fox P, Sanders H, Wright D, Vickery J, Nunn A (2003). Cannabinoids for treatment of spasticity and other symptoms related to multiple sclerosis (CAMS study): multicentre randomised placebo-controlled trial. Lancet.

[84] Pooyania S, Ethans K, Szturm T, Casey A, Perry D (2010). A randomized, double-blinded, crossover pilot study assessing the effect of nabilone on spasticity in persons with spinal cord injury. Arch Phys Med Rehabil.

[85] Wade DT, Makela PM, House H, Bateman C, Robson P (2006). Long-term use of a cannabis-based medicine in the treatment of spasticity and other symptoms in multiple sclerosis. Mult Scler.

[86] Serpell MG, Notcutt W, Collin C (2013). Sativex long-term use: an open-label trial in patients with spasticity due to multiple sclerosis. J Neurol.

[87] Notcutt W, Langford R, Davies P, Ratcliffe S, Potts R (2012). A placebo-controlled, parallel-group, randomized withdrawal study of subjects with symptoms of spasticity due to multiple sclerosis who are receiving long-term Sativex®(nabiximols). Mult Scler.

[88] Flachenecker P, Henze T, Zettl UK (2014). Nabiximols (THC/CBD oromucosal spray, Sativex®) in clinical practice—results of a multicenter, non-interventional study (MOVE 2) in patients with multiple sclerosis spasticity. Eur Neurol.

[89] Rog DJ, Nurmikko TJ, Friede T, Young CA (2005). Randomized, controlled trial of cannabis-based medicine in central pain in multiple sclerosis. Neurology.

[90] Corey-Bloom J, Wolfson T, Gamst A, Jin S, Marcotte TD, Bentley H (2012). Smoked cannabis for spasticity in multiple sclerosis: a randomized, placebo-controlled trial. CMAJ.

[91] Greenberg HS, Werness SA, Pugh JE, Andrus RO, Anderson DJ, Domino EF (1994). Short-term effects of smoking marijuana on balance in patients with multiple sclerosis and normal volunteers. Clin Pharmacol Ther.

[92] Clark AJ, Ware MA, Yazer E, Murray TJ, Lynch ME (2004). Patterns of cannabis use among patients with multiple sclerosis. Neurology.

[93] Page SA, Verhoef MJ, Stebbins RA, Metz LM, Levy JC (2003). Cannabis use as described by people with multiple sclerosis. Can J Neurol Sci.

[94] Pavisian B, MacIntosh BJ, Szilagyi G, Staines RW, O'Connor P, Feinstein A (2014). Effects of cannabis on cognition in patients with MS: a psychometric and MRI study. Neurology.

[95] Honarmand K, Tierney MC, O'Connor P, Feinstein A (2011). Effects of cannabis on cognitive function in patients with multiple sclerosis. Neurology.

[96] Rao SM, Leo GJ, Bernardin L, Unverzagt F (1991). Cognitive dysfunction in multiple sclerosis. I. Frequency, patterns, and prediction. Neurology.

[97] Leussink VI, Husseini L, Warnke C, Broussalis E, Hartung HP, Kieseier BC (2012). Symptomatic therapy in multiple sclerosis: the role of cannabinoids in treating spasticity. Ther Adv Neurol Disord.

[98] American Academy of Neurology. Position statement*:* Use of medical marijuana for neurologic disorders. Retrieved April 9, 2015, from [https://www.aan.com/uploadedFiles/Website_Library_Assets/Documents/6.Public_Policy/1.Stay_Informed/2.Position_Statements/3.PDFs_of_all_Position_Statements/Final%20Medical%20Marijuana%20Position%20Statement.pdf]

[99] Bonn-Miller MO, Vujanovic AA, Feldner MT, Bernstein A, Zvolensky MJ (2007). Posttraumatic stress symptom severity predicts marijuana use coping motives among traumatic event-exposed marijuana users. J Trauma Stress.

[100] Bremner JD, Southwick SM, Darnell A, Charney DS (1996). Chronic PTSD in Vietnam combat veterans: course of illness and substance abuse. Amer J Psychiatry.

[101] Vandrey R, Babson KA, Herrmann ES, Bonn-Miller MO (2014). Interactions between disordered sleep, post-traumatic stress disorder, and substance use disorders. Int Rev Psychiatry.

[102] Neumeister A, Normandin MD, Pietrzak RH, Piomelli D, Zheng MQ, Gujarro-Anton A (2013). Elevated brain cannabinoid CB1 receptor availability in post-traumatic stress disorder: a positron emission tomography study. Mol Psychiatry.

[103] Passie T, Emrich HM, Karst M, Brandt SD, Halpern JH (2012). Mitigation of post-traumatic stress symptoms by cannabis resin: a review of the clinical and neurobiological evidence. Drug Test Anal.

[104] Roitman P, Mechoulam R, Cooper-Kazaz R, Shalev A (2014). Preliminary, open-label, pilot study of add-on oral Δ-tetrahydrocannabinol in chronic post-traumatic stress disorder. Clin Drug Investig.

[105] Fraser GA (2009). The use of a synthetic cannabinoid in the management of treatment-resistant nightmares in posttraumatic stress disorder (PTSD). CNS Neurosci Ther.

[106] Jetly R, Heber A, Fraser G, Boisvert D (2015). The efficacy of nabilone, a synthetic cannabinoid, in the treatment of PTSD-associated nightmares: a preliminary randomized, double-blind, placebo-controlled cross-over design study. Psychoneuroendocrinology.

[107] Mashiah M. Medical cannabis as treatment for chronic combat PTSD: Promising results in an open pilot study [presentation]. Patients Out of Time Conference; Tuscon, Arizona; 2012.

[108] Cougle JR, Bonn-Miller MO, Vujanovic AA, Zvolensky MJ, Hawkins KA (2011). Posttraumatic stress disorder and cannabis use in a nationally representative sample. Psychol Addict Behav.

[109] Boden MT, Babson KA, Vujanovic AA, Short NA, Bonn-Miller MO (2013). Posttraumatic stress disorder and cannabis use characteristics among military veterans with cannabis dependence. Am J Addict.

[110] Babson KA, Boden MT, Harris AH, Stickle TR, Bonn-Miller MO (2013). Poor sleep quality as a risk factor for lapse following a cannabis quit attempt. J Subst Abuse Treat.

[111] Babson KA, Boden MT, Bonn-Miller MO (2013). The impact of perceived sleep quality and sleep efficiency/duration on cannabis use during a self-guided quit attempt. Addict Behav.

[112] Bonn-Miller MO, Boden MT, Vujanovic AA, Drescher KD (2013). Prospective investigation of the impact of cannabis use disorders on posttraumatic stress disorder symptoms among veterans in residential treatment. Psychological Trauma: Theory, Research, Practice, and Policy.

[113] Martín-Sánchez E, Furukawa TA, Taylor J, Martin JL (2009). Systematic review and meta-analysis of cannabis treatment for chronic pain. Pain Med.

[114] Phillips TJC, Cherry CL, Cox S, Marshall SJ, Rice ASC (2010). Pharmacological treatment of painful HIV-associated sensory neuropathy: a systematic review and meta-analysis of randomised controlled trials. PLoS One.

[115] Ware MA, Wang T, Shapiro S, Robinson A, Ducruet T, Huynh T (2010). Smoked cannabis for chronic neuropathic pain: a randomized controlled trial. CMAJ.

[116] Wallace M, Schulteis G, Atkinson JH, Wolfson T, Lazzaretto D, Bentley H (2007). Dose-dependent effects of smoked cannabis on capsaicin-induced pain and hyperalgesia in healthy volunteers. Anesthesiology.

[117] Wilsey B, Marcotte T, Deutsch R, Gouaux B, Sakai S, Donaghe H (2013). Low-dose vaporized cannabis significantly improves neuropathic pain. J Pain.

[118] Sallan SE, Zinberg NE, Frei E (1975). Antiemetic effect of delta-9-tetrahydrocannabinol in patients receiving cancer chemotherapy. N Engl J Med.

[119] Chang AE, Shiling DJ, Stillman RC, Goldberg NH, Seipp CA, Barofsky I (1979). Delta-9-tetrahydrocannabinol as an antiemetic in cancer patients receiving high-dose methotrexate: a prospective, randomized evaluation. Ann Intern Med.

[120] Orr LE, McKernan JF, Bloome B (1980). Antiemetic effect of tetrahydrocannabinol. Compared with placebo and prochlorperazine in chemotherapy-associated nausea and emesis. Arch Intern Med.

[121] Ben Amar M (2006). Cannabinoids in medicine: a review of their therapeutic potential. J Ethnopharmacol.

[122] Machado Rocha FC, Stefano SC, De Cassia HR, Rosa Oliveira LM, Da Silveira DX (2008). Therapeutic use of Cannabis sativa on chemotherapy-induced nausea and vomiting among cancer patients: systematic review and meta-analysis. Eur J Cancer Care (Engl).

[123] Tramèr MR, Carroll D, Campbell FA, Reynolds DJ, Moore RA, McQuay HJ (2001). Cannabinoids for control of chemotherapy induced nausea and vomiting: quantitative systematic review. BMJ.

[124] Frytak S, Moertel CG, O'Fallon JR, Rubin J, Creagan ET, O'Connell MJ (1979). Delta-9-tetrahydrocannabinol as an antiemetic for patients receiving cancer chemotherapy. A comparison with prochlorperazine and a placebo. Ann Intern Med.

[125] Ungerleider JT, Andrysiak T, Fairbanks L, Goodnight J, Sarna G, Jamison K (1982). Cannabis and cancer chemotherapy: a comparison of oral delta-9-THC and prochlorperazine. Cancer.

[126] Meiri E, Jhangiani H, Vredenburgh JJ, Barbato LM, Carter FJ, Yang HM (2007). Efficacy of dronabinol alone and in combination with ondansetron versus ondansetron alone for delayed chemotherapy-induced nausea and vomiting. Curr Med Res Opin.

[127] Lane M, Vogel CL, Ferguson J, Krasnow S, Saiers JL, Hamm J (1991). Dronabinol and prochlorperazine in combination for treatment of cancer chemotherapy-induced nausea and vomiting. J Pain Symptom Manage.

[128] Sun S, Zimmermann AE (2013). Cannabinoid hyperemesis syndrome. Hosp Pharm.

[129] Hejazi RA, McCallum RW (2011). Review article: cyclic vomiting syndrome in adults–rediscovering and redefining an old entity. Aliment Pharmacol Ther.

[130] Basch E, Prestrud AA, Hesketh PJ, Kris MG, Feyer PC, Somerfield MR (2011). Antiemetics: American society of clinical oncology clinical practice guideline update. J Clin Oncol.

[131] Roila F, Herrstedt J, Aapro M, Gralla RJ, Einhorn LH, Ballatori E (2010). Guideline update for MASCC and ESMO in the prevention of chemotherapy-and radiotherapy-induced nausea and vomiting: results of the Perugia consensus conference. Ann Onco.

[132] Gordon AJ, Conley JW, Gordon JM (2013). Medical consequences of marijuana use: a review of current literature. Curr Psychiatry Rep.

[133] Reinarman C, Nunberg H, Lanthier F, Heddleston T (2011). Who are medical marijuana patients? Population characteristics from nine California assessment clinics. J Psychoactive Drugs.

[134] Walsh Z, Callaway R, Belle-Isle L, Capler R, Kay R, Lucas P (2013). Cannabis for therapeutic purposes: patient characteristics, access, and reasons for use. Int J Drug Policy.

[135] Potter DJ, Clark P, Brown MB (2008). Potency of delta 9-THC and other cannabinoids in cannabis in England in 2005: implications for psychoactivity and pharmacology. J Forensic Sci.

[136] Hillig KW, Mahlberg PG (2004). A chemotaxonomic analysis of cannabinoid variation in cannabis (Cannabaceae). Am J Bot.

